# Type 1 Diabetes Mellitus-Related circRNAs Regulate CD4+ T Cell Functions

**DOI:** 10.1155/2022/4625183

**Published:** 2022-08-24

**Authors:** Jianni Chen, Guanfei Jia, Xue Lv, Shufa Li

**Affiliations:** ^1^Department of Endocrinology and Metabolism, The Affiliated Hospital of Qingdao University, Qingdao, 266000 Shandong, China; ^2^Department of Pharmacy, Army Medical University, Chongqing 400038, China

## Abstract

Circular RNAs (circRNAs) participate in development of malignancies through its active role as a “miRNA sponge.” Their roles in type 1 diabetes mellitus (T1DM) pathogenesis are elusive. Here, the important role of circRNAs in T1DM was explored. circRNA profiling was performed for isolated CD4+ T cells from blood of T1DM and healthy volunteers. There were 257 differentially expressed circRNAs. Only three upregulated DEcircRNAs (hsa_circ_0000324, hsa_circ_0001853, and hsa_circ_0068797) were consistent with the GEO database. Through KEGG analyses, it was found that the three DEcircRNAs were associated with 11 miRNAs and 8 immune-related target genes (mRNA). Further analysis found that four miRNAs, two circRNAs, and four mRNAs were associated with nine circRNA-miRNA-mRNA networks. This confirmed the requirements of sponge mechanisms. The qRT-PCR analysis revealed that circRNA000324/miRNA675-5p/MAPK14 and circRNA000324/miRNA-675-5p/SYK may be potential mechanisms in regulation of differentiation and proliferation of CD4+ T cell in patients with T1DM. Therefore, targeting circRNA to regulate cellular immune responses by regulating CD4+ T cell differentiation may be a new strategy for the treatment of T1DM.

## 1. Introduction

Type 1 diabetes mellitus is a condition in which pancreatic beta cells are destroyed by autoreactive T cells and insulin dysfunction [1]. Although combined antibody testing such as GADA, IA-A2, and ZnT8A has been recently performed for diagnosis of T1DM, there are patients with clinical characteristics of T1DM but negative antibodies [2]. Further, the pathogenesis of T1DM autoimmunity has not been well evaluated. Therefore, biomarkers with the potential to predict T1DM development should be investigated.

Differentiation and proliferation of autoreactive T cells (CD4+ T) is key in T1DM. Naïve CD4+ T cells can differentiate to T helper cells under stimulation by different cytokines. Biased differentiation of naïve CD4+ T into Th1 in the early stages contributes to T1DM development; subsequently, Th17 is induced to maintain the progression of T1DM. Impaired differentiation or survival of Th2 and nTregs has also been attributed to this pathological process [3–8]. It eventually activates cytotoxic T lymphocytes (CTLs) to attack *β* cells. The significance of different CD4+ T subgroups in T1DM development has been reported.

Circular RNAs (circRNAs) are crucial regulators in various biological and pathological processes, including development, apoptosis, cell differentiation, proliferation, and inflammation [9]. They are largely resistant to breakdown by exonuclease RNase [10]. circRNAs have key roles as endogenous miRNA sponges or miRNA reservoirs because some circRNAs possess miRNA response elements (MREs) that allows them to exert their inhibitory effects on and regulate target gene expressions; circRNAs function as endogenous miRNA sponges or reservoirs [11]. Moreover, circRNAs also act as transcription regulators to influence gene expression [12, 13]. Apart from their high resistance to RNase activities [10, 14], circRNAs are believed to be potential biomarkers which show organ and cell-specific expression patterns. Moreover, they are present in serum, plasma, blood, and in exosomes, hence are potential biomarkers for liquid biopsy [13, 15, 16]. Alterations in expressions of many circRNAs have been associated with various effects in autoimmune disease onset and progression [17]. Therefore, circRNAs can regulate immune functions and harbor important implications for developing therapeutic drugs [18]. A recent study involving peripheral blood mononuclear cells (PMBCs) revealed that overexpressed circPPM1F enhances M1 macrophage activation and promotes pancreatic islet injury in children with T1DM [19]. Therefore, the appearance of circRNAs that respond to differentiation and proliferation processes of CD4+ T cells is assumed to be the essential inducer for regulation of self-tolerance in T1DM.

We identified DEcircRNAs in CD4+ T cells between T1DM and normal samples by whole-transcriptome sequencing and comparisons with GEO datasets. Various bioinformatics tools were used to systematically investigate the circRNA-miRNA-mRNA interactions. The functions and associated pathways were determined through GO and KEGG pathway analyses. Then, we validated a few genes that regulate CD4+ T cell differentiation and proliferation by qRT-PCR. Our findings form the basis for identification of innovative diagnostic and drugs for T1DM.

## 2. Materials and Methods

### 2.1. Study Participants

The CD4+ T cells were isolated and extracted from diagnosed in-hospital T1DM patients on hypoglycemic therapy at the endocrinology and metabolic department of the health examination center at the affiliated hospital of Qingdao University. The control participants were healthy volunteers at the hospital. Participants were recruited from July 2019 to December 2020 (Table S1). All patients were diagnosed by clinical manifestation, islet autoantibodies (GADAb and IAA-Ab), and islet function (insulin and C peptide) assessments by at least two experienced specialists. All control participants were from the same geographic areas and had no diabetes or family history of diabetes. All potential participants with a history or family history of autoimmune disease were not included. All participants had no acute or chronic infectious diseases and were without trauma, surgery, and stress, within at least one month before blood collection. The informed consent document was obtained from patients or their families while the study was approved by the Ethics Committee of the Affiliated Hospital of Qingdao University and conducted in accordance with the principles of the Declaration of Helsinki.

### 2.2. Isolation and Extraction of CD4+ T Cells

The CD4+ T cells were isolated from peripheral venous blood samples under EDTA anticoagulation conditions. Briefly, fresh anticoagulated blood samples were diluted with the same volume of PBS (Solarbio). The diluted blood was spread on the human lymphocyte separation solution (Solarbio) to form a layered interface. It was centrifuged at room temperature (RT) for 30 min at 1000*g* and after centrifugation until stratification was formed. The white membrane layers between the plasma layer and separation solution layer were aspirated into 15 mL centrifuge tubes, centrifuged at 250*g* for 10 min after which white membrane layer cells were washed thrice using PBS. The supernatants were discarded, and cells counted for subsequent use.

After centrifugation at 300*g* for 10 min, the supernatants were completely aspirated. Cell pellets were resuspended in 80 *μ*L buffer (1 × 10^7^ cells), mixed with 20 *μ*L CD4 microbeads (Miltenyi Biotec) and incubated at 4°C for 15 minutes. Cells were washed using a buffer (auto-MACS Rinsing Solution: MACSBSA Stock Solution = 20 : 1) at 300*g* for 10 minutes and resuspended in 500 *μ*L washing buffer. The MS column was placed in the mini-MACS separator and rinsed using 500 *μ*L buffer after which the cell suspension was introduced into the column. Unlabeled cells that passed through the column were collected after which the column was washed thrice using 500 *μ*L buffer per time. The column was removed from the separator and placed on enzyme-free EP tube, and the magnetically labeled cells immediately flushed out using 1 mL buffer by firmly pushing the plunger into the column (all the products/reagents were purchased from Miltenyi Biotec). The CD4+ T cells were isolated on the same day of sample collection, immediately placed in liquid nitrogen and stored at −80°C.

### 2.3. RNA Preparation

To isolate RNA, the CD4+ T cells were treated with 2 mL TRIzol® reagent (Thermo Fisher Scientific) and then with 200 *μ*L chloroform (Merck KGaA). This was followed by vortexing for 15 sec, incubation at RT for 5 min, and centrifugation at 12,000*g* for 15 min at 4°C. We then collected the upper aqueous phase into a fresh tube and added 400 *μ*L isopropyl alcohol (Merck KGaA) to precipitate RNA. After mixing, it was allowed to stand 5 min at RT before centrifugation at 12,000*g* for 15 min at 4°C. The pellet was mixed with 1 mL 75% EtOH (Merck KGaA) and then span at 12,000*g* for 10 min at 4°C. This was followed by removal of EtOH and recentrifugation at the same conditions. The pellet was finally dried in air and then suspended in 30 *μ*L molecular grade H_2_O and treated with DNase reagent. The RNA clean-up was performed by standard EtOH precipitation.

### 2.4. Expression Profiling of RNA Microarray

RNA microarray analysis was conducted by the Beijing CNKINGBIO Biotechnology Corporation. Whole-transcriptome circRNA sequencing data from the two groups were acquired using the Illumina Hiseq2500 platform. Total RNA from CD4+ T cells of T1DM patients (*n* = 3, each consisted of a mixture of CD4+ T cells from six patients) and age-matched healthy controls (*n* = 1, mixture of CD4+ T cells from six controls) were extracted, purified, amplified, and labeled with a Low Input Quick Amp WT Labeling Kit. Labeled cRNAs were purified using the RNeasy mini kit. Each slide was hybridized with 1.65 *μ*g Cy3-labeled cRNA for 17 hours using a gene expression hybridization kit and scanned with an Agilent microarray scanner using default settings. Data were extracted using the feature extraction software 10.7 (Agilent Technologies, Santa Clara, CA, US). The Limma package in R and quantile algorithm were used to normalize the raw data. Overall, DEcircRNAs were analyzed with cutoff fold changes ≥ 2.

### 2.5. Bioinformatics Analysis and Target Prediction

The RNA microarray analysis was performed at the Beijing CNKINGBIO Biotechnology Corporation. Whole-transcriptome circRNA sequencing data were acquired from the two groups by the Illumina Hiseq2500 platform. Total RNA from CD4+ T cells of T1DM patients (*n* = 3, each consisted of a mixture of CD4+ T cells from 6 patients) and age-matched healthy controls (*n* = 1, mixture of CD4+ T cells from 6 controls) were extracted, purified, amplified, and labeled with a Low Input Quick Amp WT Labeling Kit. After labeling, the cRNA samples were treated with the RNeasy mini kit reagent to purify them. The gene expression hybridization kit was employed to hybridize the slides using 1.65 *μ*g Cy3-labeled cRNA for 17 h. The slides were analyzed with an Agilent microarray scanner under default settings, and the results were obtained with the feature extraction software 10.7 (Agilent Technologies, Santa Clara, CA, US). Raw data were normalized by the quantile algorithm and Limma package in R. Overall, we analyzed DEcircRNAs with cutoff fold changes ≥ 2.

### 2.6. qRT-PCR Validation

Only the DEcircRNAs, target miRNAs, and mRNAs that met the requirements of the sponge mechanism were validated. Their expressions were evaluated by the qRT-PCR assay using SYBR® Premix Ex Taq™ II (Takara, Japan) on a Roche 480 Real-Time PCR System. The qRT-PCR conditions consisted of an initial denaturation step of 2 min at 95°C, followed by 35 cycles of 15 s at 95°C, 10 s at 58°C, and 20 s at 72°C with fluorescence reads during extension. Melting curves (Tm) of amplicons were analyzed at 60–95°C with 0.5°C increments every 5 s. GAPDH was the internal control for mRNAs and circRNAs while U6 was the internal control for miRNAs. The 2 − ^*ΔΔ*CT^ method was conducted to analyze the PCR results. Primer sequences for qRT-PCR are shown in Table 1. Total RNAs extracted from CD4+ T cells of two groups (*n* = 30 vs. 15, T1DM vs. control) were used for validation.

### 2.7. Statistical Analysis

Normally distributed data were compared using the unpaired Student's *t*-test. *p* ≤ 0.05 was set as the threshold for statistical significance. The Pearson's correlation and linear regression were used to determine correlations. The SPSS v.19.0 and GraphPad Prism 7.0 software were used for analyses. Data are presented as mean ± SEM.

## 3. Results

### 3.1. Differentially Expressed circRNAs

**Figure 1:**
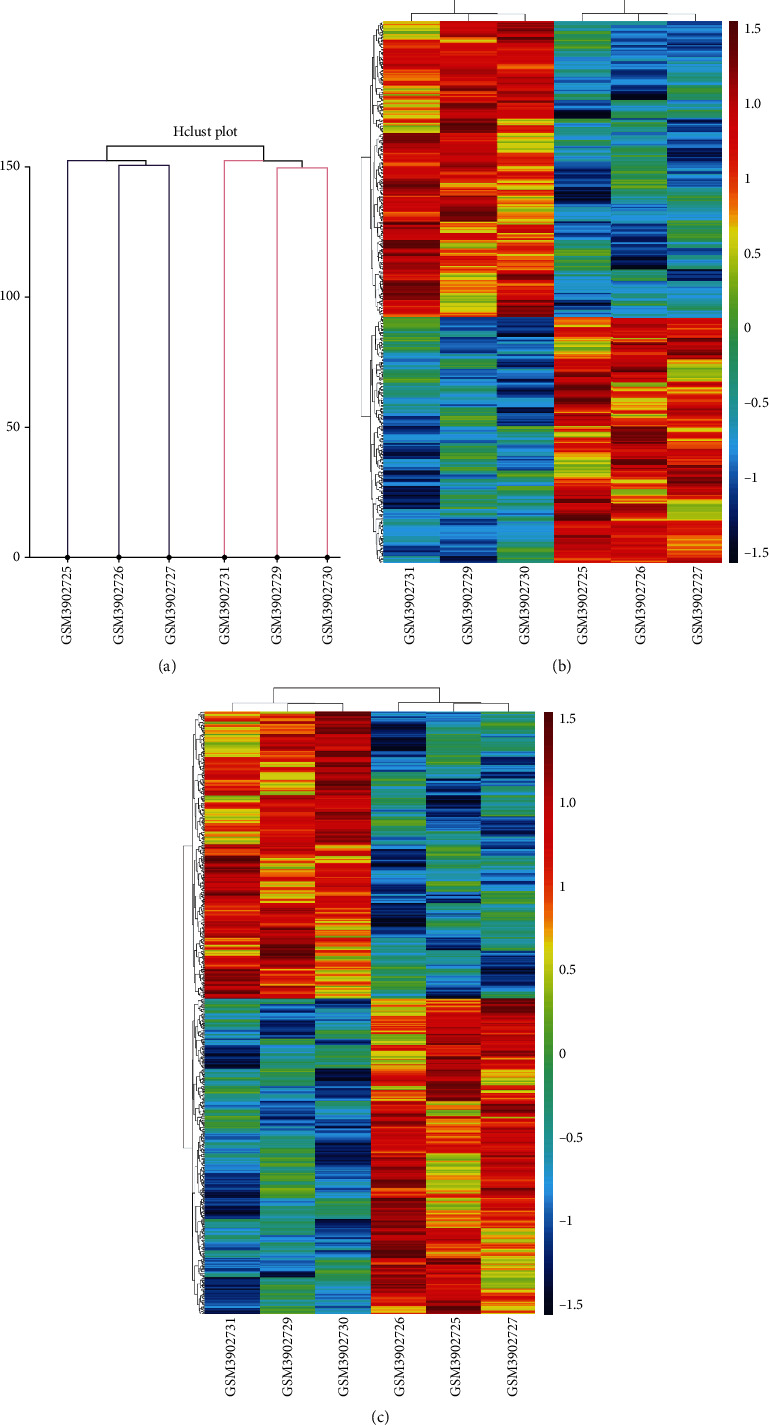
The sample clustering diagram (a) and expression profiles of circRNAs (b) and target mRNAs (c) between T1DM and control in GEO datasets GSE133225. T1DM: GSM3902729-31; control: GSM3902725-27; red strip: high relative expression; blue strip: low relative expression.

**Figure 2:**
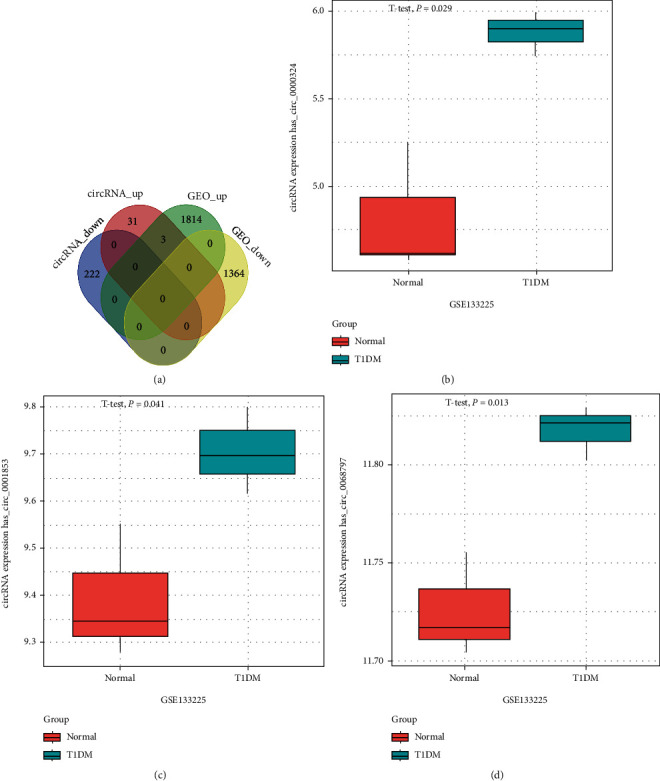
Three DEcircRNAs and their expression levels in peripheral blood mononuclear cells from T1DM patients in GEO datasets. The expression levels of hsa_circ_0000324, hsa_circ_0001853, and hsa_circ_0068797 were significantly upregulated in PBMCs from T1DM patients compared with healthy controls (*p* values < 0.05), as determined by RNA-seq.

Based on the filtration screening criteria (fold changes ≥ 2.0), it was found that there were 261 (35 upregulated, 222 downregulated, and 4 regulated in different directions among groups) DEcircRNAs in CD4+ T blood cells of T1DM patients. A total of 257 DEcircRNAs were screened for further analysis. There are 8 groups of microarray data from PBMCs of patients with T1DM and healthy volunteers in the GEO database (GSE133225), including 4 for patients with T1DM (GSM3902728, GSM3902729, GSM3902730, and GSM3902731) and 4 for normal samples (GSM3902724, GSM3902725, GSM3902726, and GSM39027). Samples GSM3902728 and GSM3902724 were excluded from the study because of poor reproducibility. Finally, 3 T1DM and 3 standardized normal sample data were obtained for identifying circRNAs (Figure 1(a)). Genes of samples with similar biological functions were clustered into one cluster. Compared to controls, circRNAs with *p* ≤ 0.05 were identified as significantly DEcircRNAs. After hierarchical clustering of these samples and their genes, there were 3182 DEcircRNAs (1365 downregulated and 1817 upregulated) between patients with T1DM and healthy controls in this database (Figure 1(b)). Intersection of 257 DEcircRNAs in our microarray data and 3182 circRNAs from the GEO database revealed 3 DEcircRNAs (hsa_circ_0000324, hsa_circ_0001853, and hsa_circ_0068797) that were significantly upregulated in both GEO databases (*p* < 0.05) (Figure 2) and our circRNA sequencing data (fold change, FC ≥ 2) as compared with the healthy controls.

### 3.2. Prediction of Target miRNAs for DEcircRNAs

**Figure 3:**
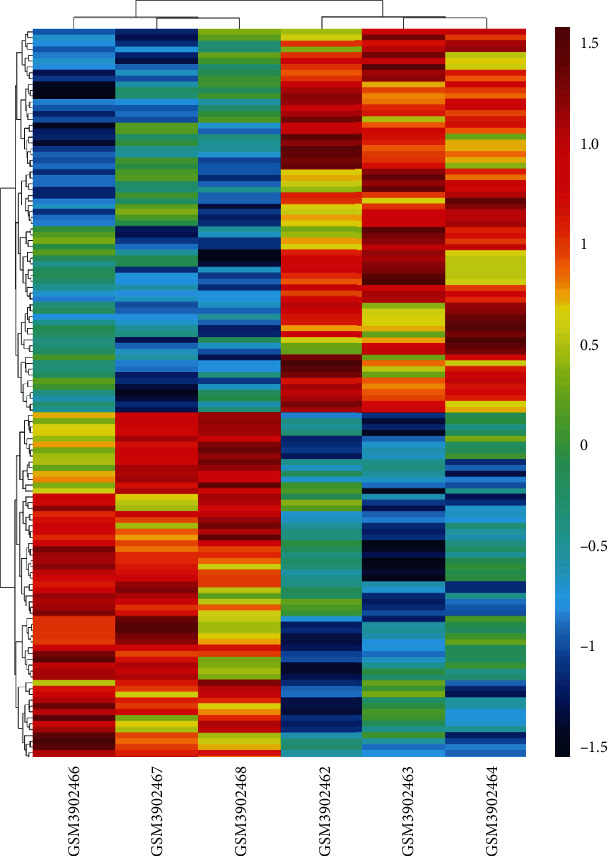
The expression profiles of miRNAs between T1DM and control in GEO datasets GSE133217. T1DM: GSM3902466-68; control: GSM3902462-64; red strip: high relative expression; blue strip: low relative expression.

**Figure 4:**
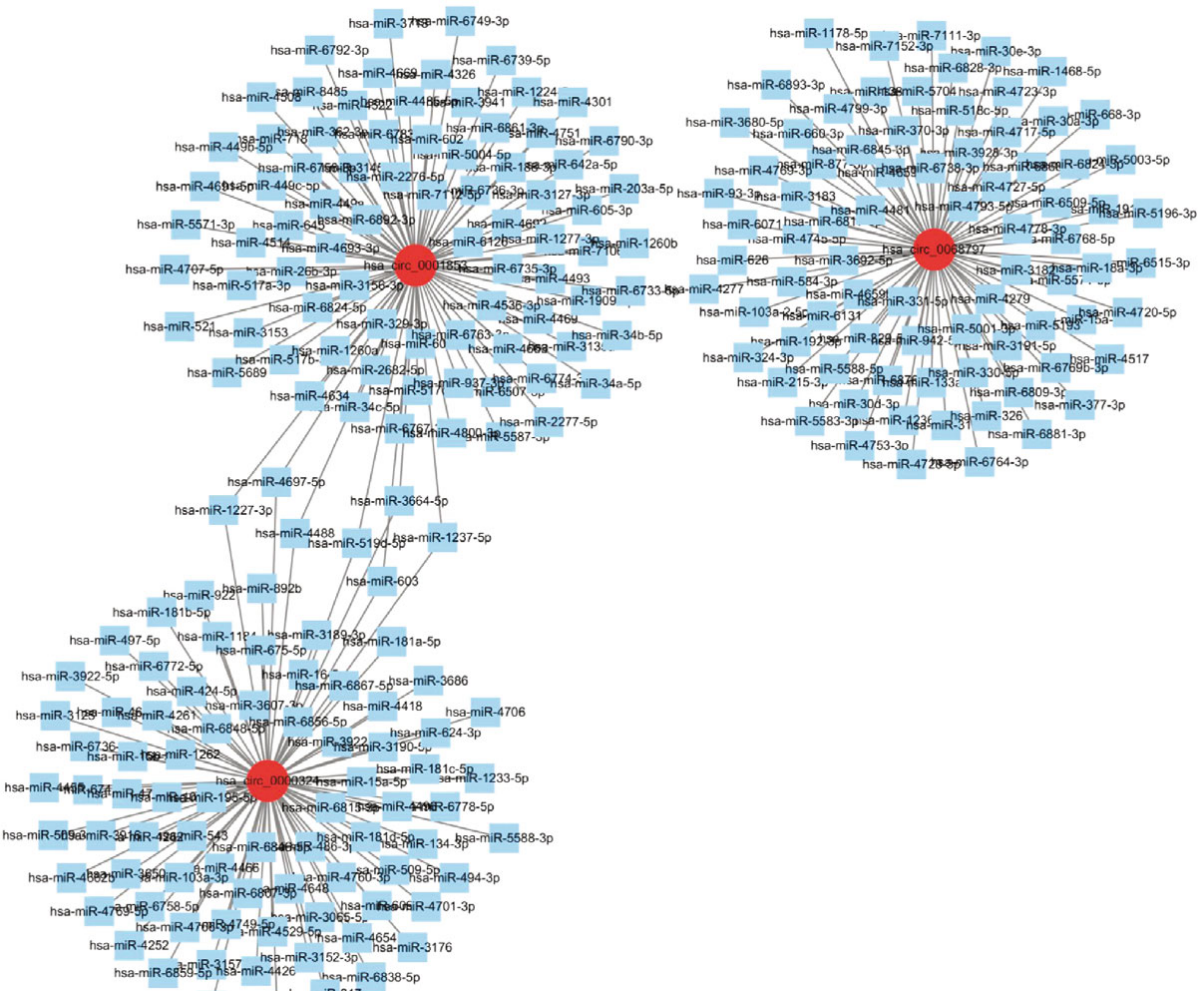
circRNA-miRNA network. Cytoscape was used to generate a circRNA-miRNA coexpression network. Node indicates the number of miRNA, indirectly representing circRNA function. Red round: circRNA; blue box: mRNA or miRNA: hsa_circ_0000324, hsa_circ_0001853, and hsa_circ_0068797 bind to 84, 88, and 89 miRNAs, respectively.

**Figure 5:**
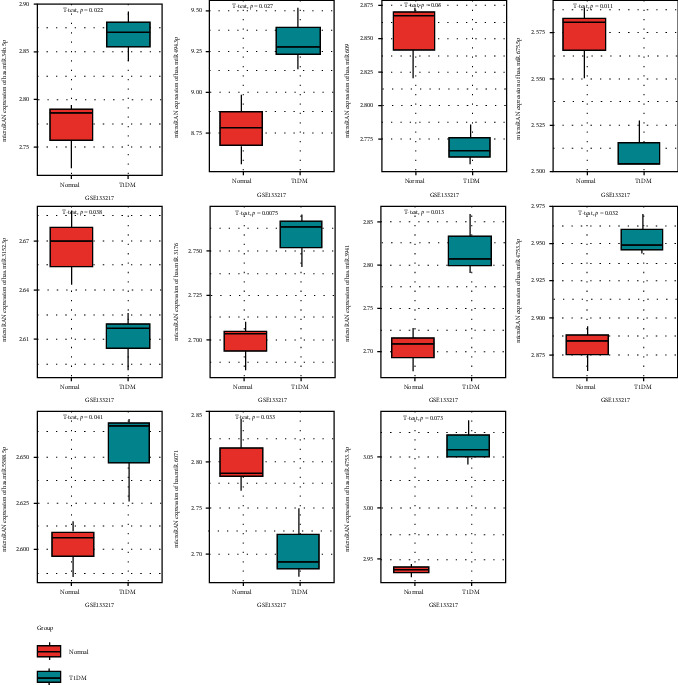
Expression levels of 11 target miRNAs in PBMCs from T1DM patients and normal controls in GEO datasets GSE133217. Red box: normal; blue box: T1DM. Seven miRNAs significantly upregulated in T1DM compared with normal controls, and four significantly downregulated.

To investigate the potential miRNAs, DEmiRNAs were obtained from the GEO database (GSE133217) based on the previously described analysis strategy. The PBMCs were obtained to perform the data of miRNAs, including 3 about the patients with T1DM (GSM3902466, GSM3902467, and GSM3902468) and 3 on the normal samples (GSM3902464, GSM3902465, and GSM3902466). The screening criteria (*p* < 0.05) revealed that 126 miRNAs were significant DEmiRNAs (Figure 2). A total of 261 miRNA targets for the three DEcircRNAs were predicted using the sequence binding algorithm in the TargetScan 6.1 software. In this study, three upregulated DEcircRNAs in CD4+ T cells were linked to the regulation of their networks by acting as miRNA sponges. Analysis of hsa_circ_000324, hsa_circ_0001853, and hsa_circ_0068797 revealed that these circRNAs separately bound to more than 80 miRNAs (84, 88, and 89, Figures 3 and 4) and hence supporting our previous hypothesis. Only 11 differentially expressed miRNAs which were possibly binding to the three DEcircRNAs were also included in the 261 target miRNAs (Figure 5).

### 3.3. Prediction of Target mRNAs for miRNAs

**Figure 6:**
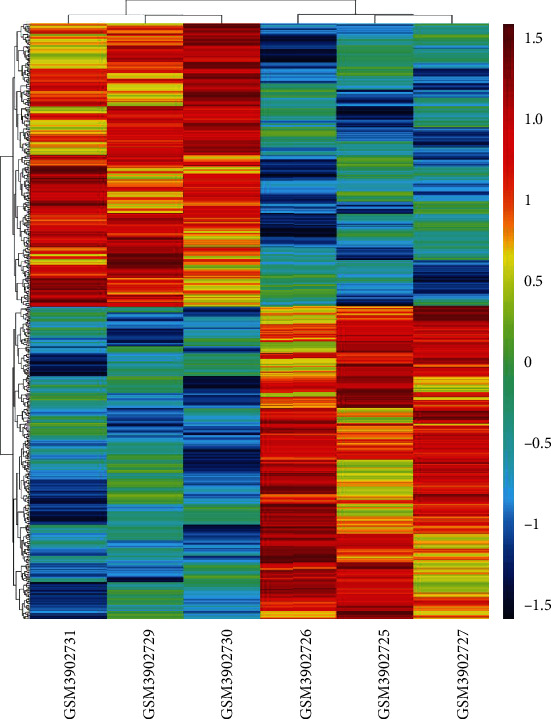
DEmRNAs from the GSE133225 (a) and the protein-protein interaction (PPI) network of predicted target genes. Nodes represent genes, and edges represent relationships between genes. The node size represents the degree, and the color from yellow to red corresponds to larger degree value (b).

**Figure 7:**
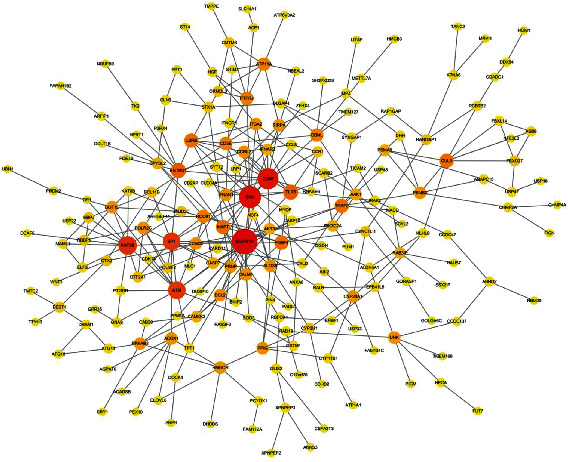
The protein-protein interaction (PPI) network of predicted target genes. Nodes represent genes, and edges represent relationships between genes. The node size represents the degree, and the color from yellow to red corresponds to larger degree value.

Only 56497 mRNAs in three or more of these databases were selected. The target mRNAs were compared with the 2349 DEmRNAs from the GEO database (GSE133225) (Figure 6). Our findings showed that there were 542 target mRNAs by intersection. Further analysis of gene interactions, which defined the “Homo sapiens” species with the minimum required interaction score as 0.4, showed that only 198 mRNAs were involved in the PPI network (Figure 7). The top 18 mRNAs, with interactional genes > 7 (degree ≥ 7), were identified as the key genes.

### 3.4. GO and KEGG Pathway Enrichment Analyses

**Figure 8:**
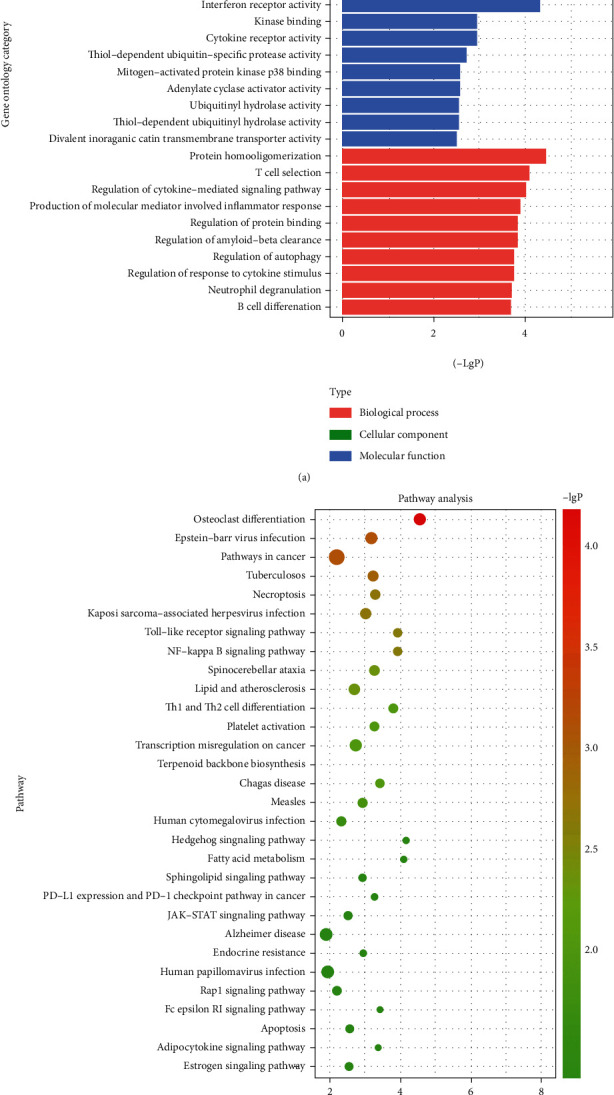
GO enrichment analysis (a) and KEGG enrichment analysis (b) for the significant functions of 542 predicted target mRNAs (*p* < 0.05).

Further, GO and KEGG analyses were carried out to determine the associated functions and pathways. Notably, 542 enriched genes were identified.  Figure 8(a) indicates that ficolin-1-rich granule lumen (GO: 1904813), ficolin-1-rich granule (GO: 0101002), ubiquitin ligase complex (GO: 0000151), and late endosome membrane (GO: 0031902) were the most enriched cellular components. For the molecular function, protein domain specific binding (GO: 0019904), interferon receptor activity (GO: 0004904), kinase binding (GO: 0019900) and cytokine receptor activity (GO: 0004896). The most enriched biological process terms were related to protein homooligomerization (GO: 0051260), T cell selection (GO: 0045058), regulation of cytokine-mediated signaling pathway (GO: 0001959), and production of molecular mediators involved in inflammatory responses (GO: 0002532).

In the KEGG analysis, 542 genes were enriched in 39 pathways. Among them, the osteoclast differentiation, Toll-like receptor, NF-kappa B, and Th1 as well as Th2 cell differentiation signaling pathways were the most enriched. The top 30 enriched pathways are shown in an enriched scatter diagram (Figure 8(b)).

To identify the key immune-related genes in T1DM and establish concise and effective networks, we excluded mRNAs that were not responsive to immune mechanisms based on literature and findings from GO and KEGG pathway analyses. Finally, 8 key mRNAs (MAPK14, SYK, CD86, ATM, TLR6, TRAF2, IL2RB, and CD3E) were selected as they were highly involved in immune-related functions and signaling pathways.

### 3.5. Prediction of circRNA-miRNA-mRNA Interaction and Network Visualization

**Figure 9:**
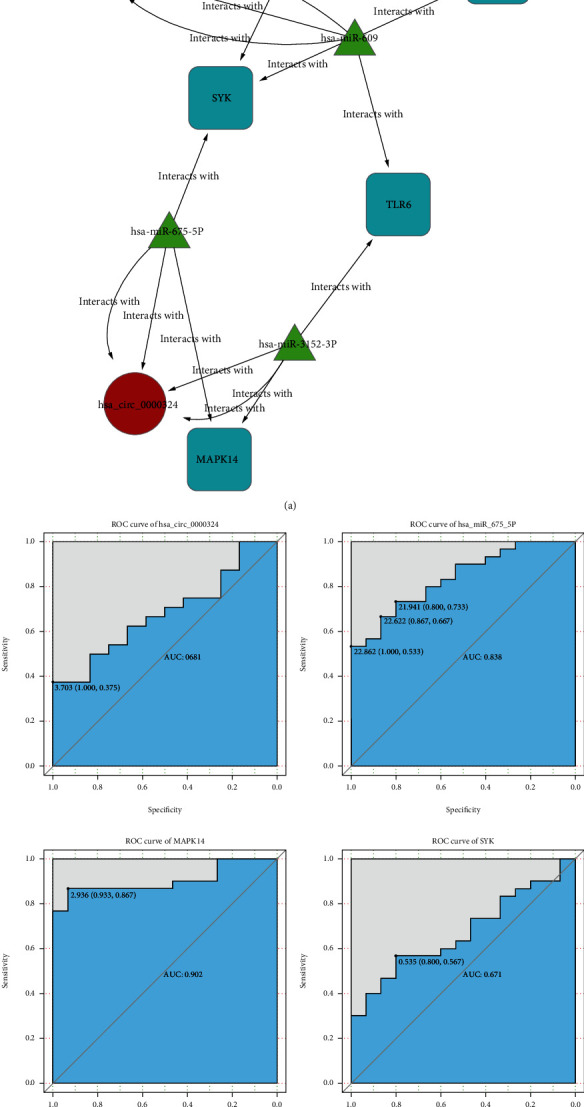
circRNA-miRNA-mRNA network (a) and validation of the expression of hsa_circ_0000324, hsa-miR-675-5p, MAPK14, and SYK in CD4+ T cells from T1DM patients and controls.

According to the spongy mechanism theory, the upregulated circRNA can lead to downregulation of miRNA and upregulated expressions of mRNA [20]. Notably, as shown in Figure 5, only four downregulated target miRNAs (hsa-miR-3152-3p, hsa-miR-6071, hsa-miR-609, and hsa-miR-675-5p) were used to construct the circRNA-miRNA-mRNA interaction network. Prediction of circRNA-miRNA interactions and networks showed only two DEcircRNAs (hsa_circ_0000324 and hsa_circ_0068797) that interacted with these four miRNAs. The miRNA-mRNA interaction prediction between these four target miRNAs and 8 immune-related key mRNAs shows that only four mRNAs (MAPK14, SYK, CD86, and TLR6) interacted with these miRNAs. Therefore, a total of eight network pathways constructed by two circRNAs (hsa_circ_0000324 and hsa_circ_0068797), four miRNAs (hsa-miR-3152-3p, hsa-miR-6071, hsa-miR-609, andhsa-miR-675-5p), and four mRNAs (MAPK14, SYK, CD86, andTLR6) were predicted (Figure 9(a)). All the ncRNAs and mRNAs in the eight pathways were validated through qRT-PCR. The result of this study showed that two circRNAs (MAPK14 and SYK) were significantly upregulated, and only one miRNA (hsa-miR-675-5p) was significantly downregulated, as compared with the controls. Finally, only the hsa_circ_0000324/miR-675-5p/MAPK14 and hsa_circ_0000324/miR-675-5p/SYK networks were successfully validated and conformed to the sponge mechanism principle (Figure 9(b)).

## 4. Discussion

circRNAs are new transcriptional and translational regulators of genes [21, 22]. Data have shown that ncRNAs play important roles in *β* cell functions, inflammation, complications, and diagnosis of T1DM [23–28]. Elsewhere, it was revealed that miR-210, miR-21, and miR-126 levels were deregulated in plasma and urinary samples of patients with T1DM [29]. circRNAs are widely distributed in different tissues, blood, and urine as well as saliva where their extractions are easy to conduct [22]. Because of their tissue and cell specificity, high-throughput sequencing of circRNAs using specific tissues and especially certain cells is the basis of accurate research, and circRNAs in body fluids are potential diagnostic markers [30, 31]. For instance, the biomarker potential of circRNAs can be increased if they were found in exosomes, as has been evidenced in solid tumors. Some circular RNAs have been shown to possess biomarker potential in colorectal cancer [32, 33].

Recently, circRNAs were reported to modulate immunocyte processes through epigenetic modifications [34–37]. Similar to other types of autoimmune diseases, T1DM is also caused by overactivation of certain immunocytes that may be regulated by circRNA. The current studies investigated the roles of circRNAs on T1DM [19, 31, 38, 39]. Two of the studies assessed human plasma circRNAs [31, 36], while one study evaluated the circRNAs of human *α*, *β*, and exocrine cells [31]. In addition, the other two studies on immunocytes were carried out on PBMCs from children with diabetes [19, 39, 40]. It has been found that circPPM1F promotes apoptosis of pancreatic *β*-cells and exacerbated pancreatic injury by promoting activation of M1 macrophage *in vitro* [19]. However, the underlying mechanisms remain unknown, and hence, there is a need for further studies to explore and elucidate it.

We investigated circRNA profiles in CD4+ T cells from T1DM patients. To improve the accuracy of bioinformatics analysis, DEcircRNA was combined with target miRNAs or mRNAs and GEO datasets for the three steps. Given the significance of CD4+ T cells in T1DM pathogenesis, it was postulated that the genes involved in the differentiation and proliferation of CD4+ T cells could be the causative genes.

To determine their functions, GO enrichment and KEGG analyses were carried out for the mRNAs. We found that the genes were presumably involved in interferon receptor activity, cytokine receptor activity, T cell selection, and regulation of cytokine-mediated signaling pathways. The results of KEGG pathway analysis showed that target mRNAs were associated with Th1 and Th2 cell differentiation pathways, NF-kappa B, and Toll-like receptor pathways. Further, the results of the prediction analysis found only two upregulated DEcircRNAs and four downregulated miRNAs, which targeted four genes. This was in accordance with the principle of sponge mechanism and coexisted in the circRNA-miRNA-mRNA coexpression network. After qRT-PCR validation, the present study found the two regulatory networks, (hsa_circ_0000324/miR-675-5p/MAPK14 and hsa_circ_0000324/miR-675-5p/SYK) which may correlate with differentiation and proliferation of CD4+ T cells in T1DM.

Collectively, the aforementioned data suggest that circRNAs are deregulated in PBMCs from T1DM patients. Further, it was evident that the hsa_circ_0000324/miR-675-5p/MAPK14 or hsa_circ_0000324/miR-675-5p/SYK network could provide a novel way to understand the disease processes and hence the prevention of T1DM. Targeting the circRNA signaling pathway to regulate CD4+ T cell functions may be an effective approach for diagnosis and treatment of T1DM. However, there is a need for further studies; more studies should be performed to verify the predicted results. Nevertheless, we demonstrate that circRNAs may be important in T1DM and hence provide a new way to investigate the circRNA functions in patients with diabetes.

This study has some limitations. First, the study evaluated circRNAs in CD4+ T cells from patients with T1DM, which differs from the sample used in the GEO dataset (PBMCs). The difference in samples may have caused the deviations in expressions of gene between the data. The CD4+ T cells from PBMCs directly reflect the immune characteristics of T1DM. Second, each group of samples in our circRNA sequence assay contained CD4+ T cells from six patients and six controls. Further, no replicate was done in control, and miRNA and mRNA microarrays were not simultaneously performed. Third, this study was based on *in vitro* and bioinformatic analyses for preliminary validation to identify multiple potential genes. There is a need for carrying out an ongoing functional experiment and *in vivo* assays to confirm our findings.

## 5. Conclusions

In conclusion, the present results reveal the circRNA profile of T1DM using circRNA-seq analyses. Further, a series of bioinformatics predicational analysis and verification experiments found the targeting miRNAs and mRNAs of DEcircRNAs. Our findings provided new evidence on the underlying mechanisms of circRNAs and related circRNA-miRNA-mRNA networks in T1DM.

## Figures and Tables

**Table 1:** Primers for qRT-PCR.

RNA ID	Primer type	Primer sequence
hsa_circ_0000324	Forward	CGGCAGGTTGGGACTTAGAT
	Reverse	GGTGTGTGCTGTGGGATAAG

hsa-miR-675-5p	Forward	CGACTCCACGACACGCACT
	Reverse	AGGGCCCACAGTGTCGTAT

MAPK14	Forward	CCCGAGCGTTACCAGAACC
	Reverse	TCGCATGAATGATGGACTGAAAT

SYK	Forward	CATGGAAAAATCTCTCGGGAAGA
	Reverse	GTCGATGCGATAGTGCAGCA

GAPDH	Forward	GGAGCGAGATCCCTCCAAAAT
	Reverse	GGCTGTTGTCATACTTCTCATGG

U6	Forward	AACGCTTCACGAATTTGCGT
	Reverse	CTCGCTTCGGCAGCACA

## Data Availability

All data are available on request from the corresponding author.

## Supplementary Materials

Supplementary Materials Table S1: clinicopathological characteristics of the patients.
