# Supplementary material for: Type 1 Diabetes Mellitus-Related circRNAs Regulate CD4+ T Cell Functions
Source: Biomed Res Int. 2022 Aug 24;2022:4625183. doi: 10.1155/2022/4625183 (PMC9433285; doi:10.1155/2022/4625183)
Supplement: Supplementary Materials — Table S1: clinicopathological characteristics of the patients. [file 4625183.f1.docx]

| **ID** | **Age** | **Course of disease** | **Gender** | **Height(cm)** | **Weight(kg)** | **GAD (IU/ml)** | **IAA (IU/ml)** | **Insulin (uiu/ml)** |
| --- | --- | --- | --- | --- | --- | --- | --- | --- |
| 1 | 55 | 9 months | female | 168 | 60 | >280.00 | 8.29 | 1.29 |
| 2 | 25 | 5 months | male | 163 | 45 | 6.25 | 18.17 | 0.96 |
| 3 | 16 | 10 days | male | 177 | 50 | 24.9 | 29.2 | 1.07 |
| 4 | 20 | 7 years | female | 160 | 62 | 63.5 | 3.48 | 13.47 |
| 5 | 33 | 20 years | female | 170 | 73 | 5.81 | 5.19 | 1.77 |
| 6 | 50 | 12 years | female | 159 | 52 | 65.01 | 20.01 | <0.40 |
| 7 | 45 | 3 days | male | 165 | 60 | 31.2 | 7.3 | 5.58 |
| 8 | 78 | 15 years | female | 156 | 53 | 121.2 | 100.9 | 4.72 |
| 9 | 63 | 40 years | female | 158 | 55 | 6.37 | 20.9 | 3.2 |
| 10 | 43 | 6 years | male | 170 | 58 | 5.89 | 2 | 1.02 |
| 11 | 51 | 17 years | male | 172 | 75 | 7.31 | 156 | <0.4 |
| 12 | 20 | 10 years | female | 165 | 60 | 5.72 | 7.81 | <0.40 |
| 13 | 59 | 20 years | female | 160 | 58 | >280.00 | 3.47 | 1.62 |
| 14 | 23 | 4 years | female | 173 | 63 | 58.89 | 7.28 | <0.04 |
| 15 | 34 | 6 years | male | 178 | 77 |  |  | 1.23 |
| 16 | 28 | 2 months | female | 160 | 40.6 | 6.29 | 2.45 | 2.15 |
| 17 | 35 | 15years | female | 158 | 70 | 7.42 | 2.58 | <0.04 |
| 18 | 20 | 10 years | male | 180 | 68 | 6.05 | 0.59 | <0.20 |
| 19 | 69 | 0.5 years | male | 158 | 47 | >280 | 3.71 | 1.49 |
| 20 | 34 | 10 years | male | 172 | 61.6 | 6.9 | 15.1 | 1.11 |
| 21 | 20 | 4 days | female | 152 | 40 | 57.03 | 3.12 | 1.42 |
| 22 | 51 | 6 years | male | 162 | 44 | 9.5 | 5.43 | 1.01 |
| 23 | 31 | 4 years | male | 174 | 57 | 77.4 | 18.1 | 1.99 |
| 24 | 20 | 10 years | female | 165 | 60 | 5.72 | 7.81 | <0.40 |
| 25 | 49 | 21 years | male | 178 | 68 | 23.3 | 8.7 | <0.40 |
| 26 | 23 | 1 years | male | 178 | 60 | 90.28 | 2 | <0.4 |
| 27 | 63 | 2 years | female | 165 | 51.5 |  |  | 1.96 |
| 28 | 15 | 0.5 years | female | 167 | 60 | 6.12 | 2.89 | 1.67 |
| 29 | 34 | 11 years | female | 165 | 76 | 7.89 | 14.94 | 7.51 |
| 30 | 43 | 8 years | male | 168 | 67 | 87 | 95 | 3.26 |

**Table S1** Clinicopathological characteristics of the patients

| **ID** | **INS (uIU/ml**）1h | **INS (uIU/ml**）2h | **C-Peptide(ng/ml)** | **C-Peptide(ng/ml) 1h** | **C-Peptide(ng/ml) 2h** | **HbA1c (%)** |
| --- | --- | --- | --- | --- | --- | --- |
| 1 | 2.76 | 1.21 | 0.4 | 0.59 | 0.38 | 11.3 |
| 2 | 0.86 | 0.47 | 5.04 | 0.86 | 2.44 | 4.9 |
| 3 | 1.83 | 2.43 | 0.4 | 0.46 | 0.65 | 5.3 |
| 4 | 0.45 | <0.40 | <0.01 | <0.02 | <0.02 | 12 |
| 5 | 0.45 | <0.40 | <0.02 | <0.02 | <0.02 | 8 |
| 6 |  | <0.40 | <0.02 |  | <0.02 | 8 |
| 7 | 4.76 | 2.62 | 0.57 | 0.5 | 0.53 | 15.9 |
| 8 | 12.3 | 5.17 | 0.12 | 0.9 | 0.33 | 8.3 |
| 9 | 2.06 | 2.04 | 0.19 | 0.2 | 0.21 | 6.4 |
| 10 | 1.76 | 1.67 | 0.4 | 0.63 | 0.63 | 9.6 |
| 11 | <0.40 | <0.40 | <0.02 | <0.02 | <0.02 | 8.4 |
| 12 |  |  | 0.12 |  |  | 14.1 |
| 13 | 1.75 | 1.88 | <0.02 | 0.03 | 0.02 | 8.3 |
| 14 | <0.04 | <0.04 | <0.02 | <0.02 | <0.02 | 9.8 |
| 15 | 2.44 | 2.24 | 0.3 | 0.53 | 0.65 | 10.9 |
| 16 | 5.68 | 3.62 | 0.56 | 0.85 | 0.75 | 11.8 |
| 17 | <0.40 | <0.40 | <0.02 | <0.02 | <0.02 | 10.3 |
| 18 | <0.20 | <0.20 | 0.1 | 0.2 | 0.17 | 7.2 |
| 19 | 2.19 | 1.05 | 0.66 | 0.68 | 0.52 | 9.9 |
| 20 |  |  | <0.02 |  |  | 10.3 |
| 21 | 1.42 | 1.22 | 0.21 | 0.38 | 0.44 | 16.1 |
| 22 | 1.46 | 0.89 | 0.56 | 0.57 | 0.45 | 11.7 |
| 23 | 0.64 | 0.58 | 0.05 | 0.05 | 0.03 | 7.6 |
| 24 |  |  | 0.12 |  |  | 14.1 |
| 25 | <0.40 | <0.40 | <0.02 | <0.02 | <0.02 | 9.2 |
| 26 |  |  | 0.39 |  |  | 15.33 |
| 27 |  |  | 0.78 |  |  | 10.4 |
| 28 | 4.28 | 5.91 | 0.64 | 0.92 | 1.14 | 15.6 |
| 29 | 17.9 | 6.58 | 2 | 2.4 | 2.11 | 10.8 |
| 30 | 76.3 | <0.40 | <0.02 | <0.02 | <0.02 | 9.4 |
